# An Open-Source Cognitive Test Battery to Assess Human Attention and Memory

**DOI:** 10.3389/fpsyg.2022.880375

**Published:** 2022-06-10

**Authors:** Maxime Adolphe, Masataka Sawayama, Denis Maurel, Alexandra Delmas, Pierre-Yves Oudeyer, Hélène Sauzéon

**Affiliations:** ^1^Flowers Team, Inria, Bordeaux, France; ^2^Research and Development Team, Onepoint, Bordeaux, France; ^3^Department of Cognitive Sciences and Ergonomics, Université de Bordeaux, Bordeaux, France; ^4^Microsoft Research Montreal, Montreal, QC, Canada; ^5^ACTIVE Team, Université de Bordeaux, INSERM, BPH, U1219, Bordeaux, France

**Keywords:** online experiment, multiple object tracking (MOT), enumeration, load induced blindness, go/no-go decision, task-switching, working memory, memorability

## Abstract

Cognitive test batteries are widely used in diverse research fields, such as cognitive training, cognitive disorder assessment, or brain mechanism understanding. Although they need flexibility according to their usage objectives, most test batteries are not available as open-source software and are not be tuned by researchers in detail. The present study introduces an open-source cognitive test battery to assess attention and memory, using a javascript library, p5.js. Because of the ubiquitous nature of dynamic attention in our daily lives, it is crucial to have tools for its assessment or training. For that purpose, our test battery includes seven cognitive tasks (multiple-objects tracking, enumeration, go/no-go, load-induced blindness, task-switching, working memory, and memorability), common in cognitive science literature. By using the test battery, we conducted an online experiment to collect the benchmark data. Results conducted on 2 separate days showed the high cross-day reliability. Specifically, the task performance did not largely change with the different days. Besides, our test battery captures diverse individual differences and can evaluate them based on the cognitive factors extracted from latent factor analysis. Since we share our source code as open-source software, users can expand and manipulate experimental conditions flexibly. Our test battery is also flexible in terms of the experimental environment, i.e., it is possible to experiment either online or in a laboratory environment.

## 1. Introduction

Cognitive abilities such as attention or memory are essential for our daily life. Researchers have measured these abilities for many years to elucidate human cognitive mechanisms, diagnose various mental disorders, and evaluate cognitive training effects. Previous studies in cognitive science have generally investigated a specific task using various stimulus parameters to understand the underlying mechanisms in detail (Luck and Vogel, 1997; Baldauf and Desimone, 2014; Maunsell, 2015). On the other hand, works in the cognitive diagnosis and training literature utilize test batteries, including various cognitive tasks, to evaluate the individual's diverse cognitive state (Green and Bavelier, 2003; Kueider et al., 2012; Lampit et al., 2014; Hosokawa et al., 2019; Steyvers and Schafer, 2020). For instance, researchers in cognitive training studies leverage a cognitive test battery before and after training to estimate how their intervention affects the cognitive state. Since the purpose of cognitive test batteries generally needs to cover a variety of cognitive abilities, such as vision, memory, auditory, or logical reasoning (Folstein et al., 1975; Nasreddine et al., 2005), each task includes a small number of stimulus parameters to keep the experimental time short. However, if researchers focus on specific cognitive abilities in cognitive training investigations, e.g., visual attention or memory, such limited parameters can be insufficient to evaluate cognitive states because complicated cognitive processes mediate each ability, as explored in the cognitive mechanism investigations.

The present study aims to develop an online open-source test battery to leverage the two research directions, i.e., cognitive mechanism understanding and test battery assessment. Specifically, while investigating various parameters for each task, as in the investigation of cognitive mechanisms, we have explored the relationship across diverse cognitive tasks, as in the studies of test batteries. We consider that one of the difficulties in developing such extension in the previous literature is mainly related to the proprietary nature of existing cognitive assessment software. Indeed, either classic or computerized, most cognitive batteries are commercial (Conners et al., 2000; Kraus and Breznitz, 2009; Preiss et al., 2013; Mielke et al., 2015; Hosokawa et al., 2019) and the researchers do not have flexible control over the parameters of the program. While this constraint allows researchers to share a common standard framework, it does not easily allow the work to be extended to new goals. Since the trend of experimental environments quickly changes depending on the technology development, flexibility and openness of the software are essential to ensure that the test battery is used over a long period. For instance, there has recently been a great demand to investigate online experiments. Some recent cognitive training studies also utilize online training. To evaluate the effect of such training, one needs to evaluate the cognitive ability using an online test battery. Since our test battery uses a browser-based platform, using a javascript library, p5.js, experimenters can flexibly launch the experiment under various environments referring to its source code.

Our test battery includes seven cognitive tasks: multiple object tracking, enumeration, load-induced blindness, go/no-go, task switching, working memory, and memorability. Our purpose is to create a test battery for cognitive training studies that focuses on the specific capacity of visual attention and memory rather than multiple cognitive domains such as auditory, linguistic, and logical reasoning tasks, as in previous work (Steyvers and Schafer, 2020; Soreq et al., 2021). In particular, we selected tasks related to a multiple object tracking (MOT) task measuring dynamic attention (Culham et al., 1998; Cavanagh and Alvarez, 2005). MOT is a cognitive task in which participants are required to track multiple moving objects simultaneously in a cluttered scene. Because such tracking abilities are essential in daily situations, many cognitive training works utilize MOT as a training task (cf. Vater et al., 2021) for various participant populations, such as young adults (Legault and Faubert, 2012; Harris et al., 2020), older adults (Legault and Faubert, 2012; Legault et al., 2013), professional athletes (Faubert, 2013), and video game players (Benoit et al., 2020). For instance, Legault et al. (2013) used a 3D MOT task for training and showed that the training efficiency for healthy older adults was similar to younger adults. Based on a task related to object tracking abilities, we used the taxonomy of Barnett and Ceci (2002) to build the assessment tool. On the content dimension, we chose the enumeration and load-induced blindness tasks for the near transfer tasks related to MOT, as some previous work has shown their performance correlation (Green and Bavelier, 2006b; Eayrs and Lavie, 2018). For the far transfer tasks, we used other attention tasks, i.e., go/no-go and task switching tasks. On the memory dimension, as some have shown the contribution of working memory abilities to MOT performance (Allen et al., 2006; Lapierre et al., 2017), we used spatial working memory and memorability tasks as near and far transfer tasks, respectively. The choice of these tasks also allows for the evaluation of a transfer on the dimensions of the type of outcomes (e.g., accuracy, reaction time) as well as on the specificity (e.g., single and dual tasks) of the tasks. Because cognitive training studies can use our test battery as a pre/post evaluation, we consider that each participant can complete all tasks within an hour and a half.

**Figure 1:**
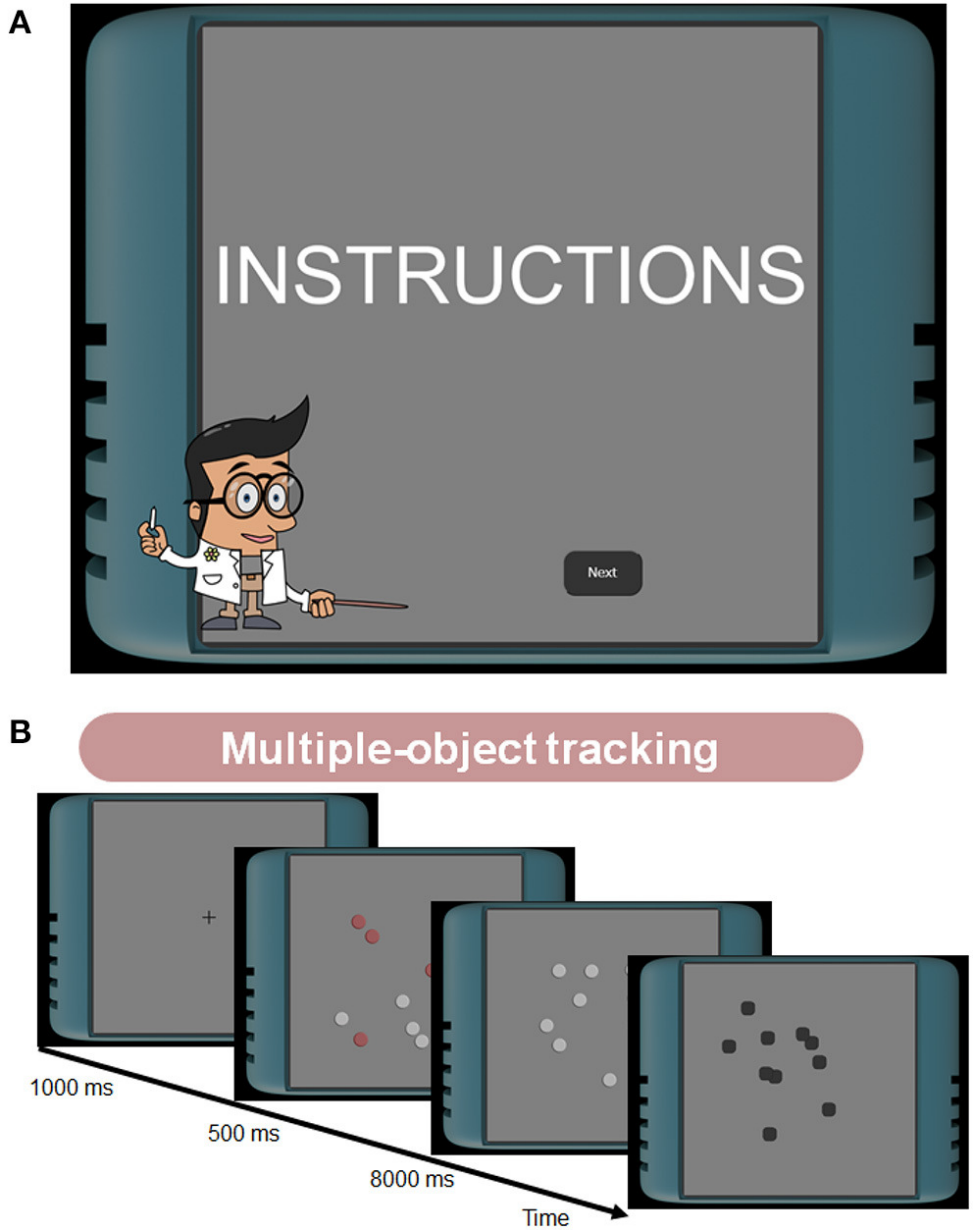
**(A)** Interface example of the test battery. **(B)** Stimuli and tasks in the multiple-object tracking (MOT) task. After presenting the fixation point, five of the 10 discs were briefly highlighted in red to show they are the targets to track. Then, 10 objects started to move for 8 s. Participants answered which are the target objects by clicking black boxes after the moving scene ended.

**Figure 2:**
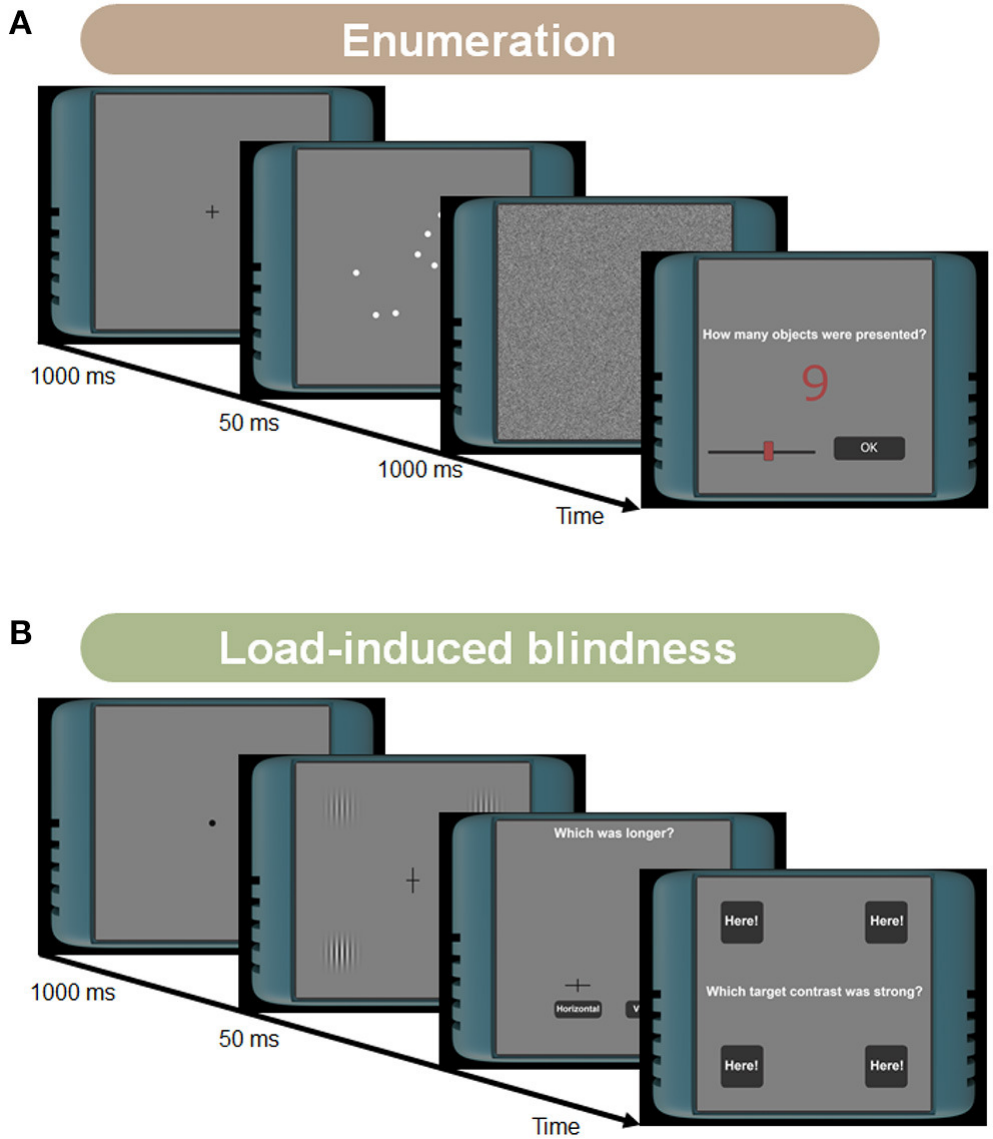
**(A)** Stimuli and tasks in the enumeration task. After presenting the fixation point, a brief flash of multiple white circles was presented. Participants answered how many circles were shown by using a slider. **(B)** Stimuli and tasks in the load-induced blindness task. Participants were asked to perform a dual-task, answering the length of the gazing point and the contrast of the images presented in the surroundings. After showing the fixation dot, a cross target with four Gabor stimuli was briefly presented. Participants first answered which of the lines was longer using mouse clicking. Then they answered which of the four Gabor stimuli had the enhanced contrast by clicking one of the four buttons.

**Figure 3:**
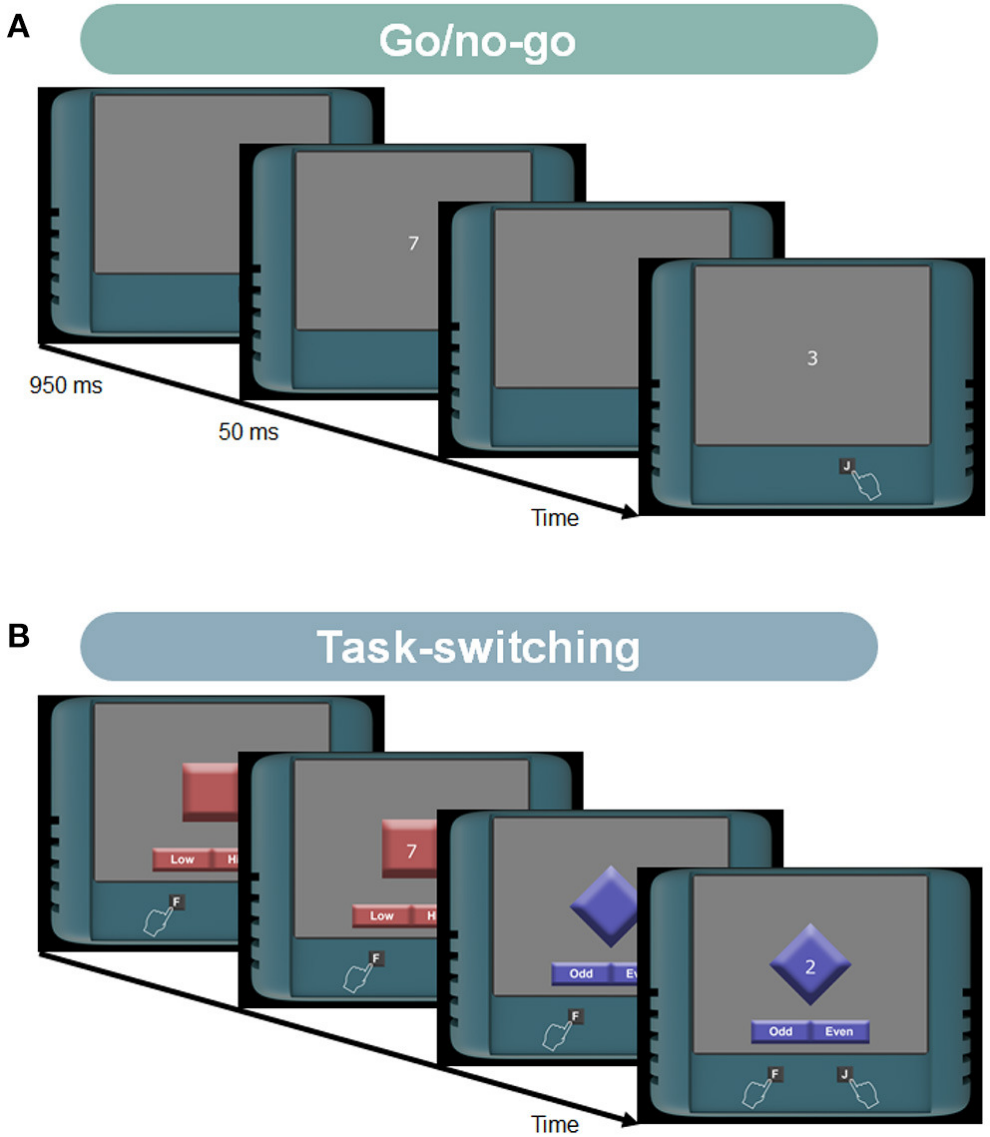
**(A)** Stimuli and tasks in the go/no-go task. On each trial, a digit was briefly presented one by one. Participants were asked to focus on the number “7” and answer whether the number after the “7” was the “3” or not. If the number after “7” was “3,” they had to press the key “J” as soon as possible. **(B)** Stimuli and tasks in the task-switching task. Participants' tasks changed with the task cue. When the task cue was the blue diamond-shaped background, participants had to answer whether the target digit was odd/even. In contrast, when the task cue was the red square background, they had to answer whether the target digit was higher/lower than five.

**Figure 4:**
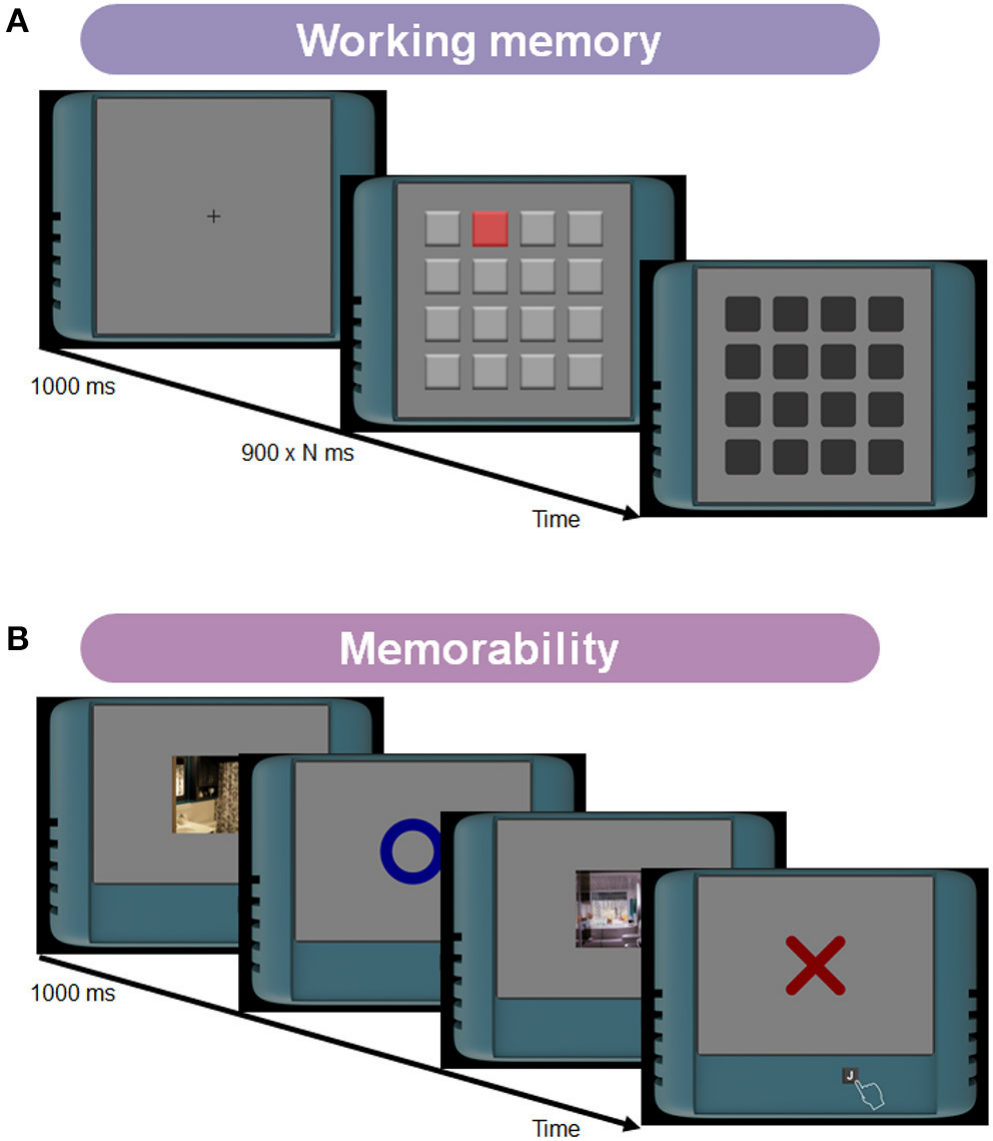
**(A)** Stimuli and tasks in the working memory task. On each trial, one of sixteen squares was sequentially flashed with a reddish color briefly. After the presentation, participants answered the sequence by clicking on the squares in the same order. **(B)** Stimuli and tasks in the memorability task. On each trial, a natural scene photograph was presented one by one. Participants were asked to remember each photograph and answer whether the photograph is presented twice or not.

The tasks have been intensively investigated in visual attention and memory literature. The multiple-object tracking task measures participants' tracking ability (Figure 1B; Pylyshyn and Storm, 1988; Cavanagh and Alvarez, 2005; Bettencourt and Somers, 2009; Vul et al., 2009; Zhong et al., 2014). The task difficulty depends on multiple factors such as the number of targets, the number of distractors, or the object speed. The enumeration task measures human counting ability (Figure 2A; Trick and Pylyshyn, 1993; Green and Bavelier, 2003, 2006b). This task asks participants to count flashed multiple objects. Depending on the counting number, it has been known that observers show different cognitive performances. Specifically, for smaller numbers of items (e.g., 2–4), participants can count them effortlessly and quickly, as called “subitizing.” In contrast, it has been considered that the counting efficiency decreases for larger numbers of items (e.g., more than 5), which we used in our experiments. The load-induced blindness task measures the divided attention and the useful field of view (Figure 2B; Macdonald and Lavie, 2008; Dye and Bavelier, 2010; Eayrs and Lavie, 2018). This task asks participants to perform a dual-attention task, both foveal and peripheral detection tasks. Since this task requires peripheral target detection, it is related to the works of the useful field of view (UFOV) (Edwards et al., 2018). The UFOV is generally hard to measure using online experiments because it needs a large visual angle. However, using a dual-task that needs divided attention can narrow the field. The go/no-go task measures the ability to distinguish between relevant and irrelevant information (Figure 3A; Conners et al., 2000; Bokura et al., 2001; Mani et al., 2005; Nash et al., 2013). This task requires participants to attend a cue and to answer if the following stimulus is the target or not. This task is also known as a standard cognitive test, called the cued continuous performance test (CPT) (Conners et al., 2000). The task-switching task measures the flexibility of selective attention (Figure 3B; Meiran, 1996; Monsell, 2003; Monsell et al., 2003). This task requires participants to shift their attention between different goals. A lot of paradigms have been suggested to measure flexibility (Monsell, 2003). We chose the task-cueing paradigm in the task-switching paradigms, where the task was unpredictable, and a task cue appeared before and with the stimulus (Monsell et al., 2003) because it is easier to present to online participants. The working memory task here indicates the spatial span task (the Corsi Block Tapping Task) and is to measure spatial short-term memory capacity (Figure 4A; Berch et al., 1998; Soreq et al., 2021).

Another advantage of our test battery is that we compare a recent cognitive task, the memorability task, with traditional ones used in the test battery literature. The memorability task measures the ability to memorize natural scene images (Figure 4B; Isola et al., 2011; Bylinskii et al., 2015, 2021; Khosla et al., 2015). This task was initially proposed in the computer vision community to explore what intrinsic image features are memorable for human participants. However, not only the intrinsic image factors but also human cognitive factors mediate this task performance. Specifically, even if an image has identical intrinsic factors, memorability can change with how observers pay attention to it (Mancas and Le Meur, 2013; Bylinskii et al., 2015). Although some studies show the contribution of cognitive factors to memorability, it is still unclear how it relates to diverse human cognitive abilities. Thus, the inclusion of the memorability task to our test battery can contribute to either cognitive mechanism understanding or diverse test battery development. For the cognitive mechanism understanding, our investigation can clarify what kind of underlying cognitive abilities mediate memorability performance by comparing other cognitive task performances. Moreover, for the test battery development, including a cognitive task using natural scene images is needed to assess the ecological validity of cognitive training because most tests use artificial stimuli. The difficulty of using natural scene images is how to control the task difficulty as it has to be controlled constantly using different natural images. An advantage of the memorability datasets is that the previous works of the memorability share the experimental data (Isola et al., 2011; Bylinskii et al., 2015, 2021; Khosla et al., 2015), and we can extract constant difficulty images from the datasets.

In the following Section 2, we describe each task in detail and how to collect the benchmark data. After discussing our benchmark data in Sections 3, 4, we show the data availability. In summary, our investigation includes the following features:

1. We suggest an online open-source cognitive test battery including a wide variety of attention and memory tasks with various stimulus parameters.
2. Experimenters can flexibly run it in various environments (online or in the laboratory) using a web browser.
3. Our test battery captures diverse individual differences and can evaluate them based on the latent cognitive factors.
4. It is flexible in expanding stimulus conditions and adding new tasks because all source codes and data are available. Besides, we prepare a playground to test our cognitive tasks to support users' understanding of the task procedure in the following link (https://github.com/flowersteam/cognitive-testbattery).

## 2. Materials and Methods

### 2.1. Participants

Fifty naïve participants, aged from 21 to 71 (median = 25, mean = 29.0, standard deviation = 11.6) years old, engaged in the experiments. All gave informed consent approved by the Operational Committee for the Evaluation of Legal and Ethical Risks (OCELER).

### 2.2. Apparatus

The benchmark data acquisition was conducted online using a web browser with a javascript library, p5.js (https://p5js.org/). Each participant accessed the web server hosted in our laboratory and engaged in the tasks. The platform in our experiment was organized by a python web framework, Django (https://www.djangoproject.com/). The informed consent and the schedule management of the 2-days experiment were controlled using the platform. Our test battery, including instructions and practice trials for each task, was implemented with the javascript library p5.js. Users can run our test battery either with our Django platform or separately using a shared webserver. All codes and data are available from the following link: https://github.com/flowersteam/cognitive-testbattery.

### 2.3. General Procedure

Our experiment has been conducted over 2 days (median time between pre and post assessment: 1 day and 16 h). Participants registered for the experiment on the first day and reported the monitor size in cm or inch. We asked them to use the same monitor across days and to see the monitor from a distance of 50 cm. We extracted the monitor pixel size they used and defined the visual angle based on the information, as common in online experiments (Li et al., 2020). During the experiment, a virtual character provided the guidelines on how to use the experimental platform (Figure 1A). Including the character, the platform was implemented as a gamified interface to keep participants' motivation high (Clement et al., 2013; Lumsden et al., 2016; Hosokawa et al., 2019).

### 2.4. Stimuli and Procedure of Each Task

This subsection describes the stimuli and procedure of each task. We decided the stimulus parameters of each task based on previous cognitive science works and our preliminary investigation on a browser-based investigation.

#### 2.4.1. Multiple-Object Tracking Task

Our task procedure followed Bettencourt and Somers (2009) because it fits to conduct online experiments efficiently while exploring the tracking ability for the numbers of targets and the target speed conditions (Figure 1B). In our experiment, either the target or distractor number was five. We controlled the task difficulty by changing the target speed in 1, 4, and 8 degrees/s. The diameter of each disk was 1.2°. On each trial, five of the ten discs were briefly highlighted in red for 1 s to show they were the targets to track. After that, ten objects started to move for 8 s. The moving direction was determined randomly at first and bounced at the corner of the square canvas of 12 × 12 degrees. We allowed the occlusion between the objects. Participants' task was to remember the target discs and track these positions until they stopped. They answered the target position by clicking the buttons placed on the final object positions.

#### 2.4.2. Enumeration Task

This task procedure followed Green and Bavelier (2006b) in which the authors compared an enumeration task with the ability of MOT (Figure 2A). Each trial began with the presentation of a fixation pattern in the center of a middle gray background. After 1,000 ms, a set of white circles was presented for 50 ms. The number of circles was selected from 5, 6, 7, 8, or 9 in a pseudo-random order. The diameter of each circle was 0.5 × 0.5°, and its color was white. The circles were presented within a diameter of 5° in the background. The position of the circles was not overlapped in the region. The participants were asked to count these circles and answer how many circles were presented using a slider bar. Each stimulus condition was tested 20 times for each observer.

#### 2.4.3. Load-Induced Blindness Task

The load-induced blindness procedure followed Eayrs and Lavie (2018), in which the load-induced blindness ability was compared with MOT (Figure 2B). On each trial, after presenting the fixation pattern of 1,000 ms, participants viewed a 50 ms presentation of a cross target with four Gabor stimuli. After 950 ms, they were asked to answer which of the lines was longer using mouse clicking. Then, they were asked to answer which of the four Gabor stimuli had the enhanced contrast by clicking one of the four buttons. They were asked to correctly answer at least the foveal task. If not, their response to the peripheral task was not recorded. The length of each cross pattern was 0.5 or 1.0°, and the vertical or horizontal line was randomly selected for the longer one. The color of the cross was black. For the Gabor stimuli, the standard deviation of the Gaussian envelope was 0.7°. The spatial frequency and the orientation of the grating were 2.2 cycles/degree and 0.0 degrees, respectively. The mean luminance was set to the background color (i.e., middle gray). The enhanced contrast of the target was 0.8, and the others were 0.4. The Gabor stimuli were presented at a distance of 3 (near condition) or 6 (far condition) degrees from the center position on the screen. Each stimulus condition was tested 20 times for each observer.

#### 2.4.4. Go/No-Go Task

The ten single digits (from 0 to 9) were used as the stimuli (Figure 3A). We decided the stimulus presentation time based on Mani et al. (2005), though we cannot strictly control the presentation time due to browser-based experiments. Each trial began with the 1,000 ms presentation of the fixation point. Then, each digit was presented one by one for 50 ms with the interstimulus interval (ISI) of 950 ms. The digit stimuli were presented within an area of 1.5 degrees squares. Participants were asked to focus on the number “7” and answer whether the number after the “7” was the “3” or not. If the number after “7” was “3” (Go trial), they had to press the key “J” as soon as possible. If not (No-go trial), they were asked to keep not responding. The probability of Go/No-go trials was 50/50%. Each stimulus condition was tested 18 times for each observer.

#### 2.4.5. Task-Switching Task

We used the task-cueing paradigm in the task-switching paradigms, where the task was unpredictable, and a task cue appeared before and with the stimulus (Figure 3B) (Monsell et al., 2003). We designed our original stimulus patterns to make the cues and tasks clearer for online participants. A digit from the set 1–4, 6–9 was used for the target stimuli. Participants' tasks changed with the task cue. When the task cue was the blue diamond-shaped background, participants had to answer whether the target digit was odd/even by using the key “F”/“J,” respectively. In contrast, when the task cue was the red square background, they had to answer whether the target digit was higher/lower than five by using the key “F”/“J,” respectively. Each trial began with the presentation of the task cue. After the cue presentation of 650 ms, a target digit was displayed. The size of the background rectangle was 4.9° on each side. The target digit was shown in the center of the background within an area of 1.5° squares. After the participant's response, a blank screen of 1,000 ms was presented. We used the first 20 trials as practice ones. Each stimulus condition was tested 30 times for each observer.

#### 2.4.6. Working Memory Task

We used a typical procedure of computerized Corsi Block Tapping tasks (Figure 4A) (e.g., Soreq et al., 2021). On each trial, sixteen light gray squares were displayed in a four-by-four grid. One of these squares was sequentially flashed with a reddish color for 900 ms. The order of the flashes was randomized for each trial. After the flash presentation, participants were asked to repeat the sequence by clicking on the squares in the same forward order. The size of each square was 2.0° on each side. The number of flashes was selected from the set of {4,5,6,7,8} in a pseudo-randomized order. Each stimulus condition was tested 12 times for each observer.

#### 2.4.7. Memorability Task

The memorability task measures human memory performance for natural scene images (Figure 4B; Isola et al., 2011; Bylinskii et al., 2015, 2021; Khosla et al., 2015). Our experiment extracted images with intermediate memorability scores from the FIne-GRained ImageMemorability (FIGRIM) dataset (Bylinskii et al., 2015) because it has been shown that cognitive factors are more effective for these images. Each trial began with the presentation of a natural scene photograph for 1000 ms. During the presentation, participants were asked to remember each photograph and answer whether the photograph is presented twice or not, by pressing the key “J” as soon as possible. After each presentation, participants received feedback if the response was correct or not for 1,400 ms. There were two blocks for the memorability task. On each block, participants viewed a set of 120 images within a specific natural scene category, “bedroom” or “kitchen,” in the dataset. We chose the image of hit rates on the interval [0.60, 0.70]. Forty images were the targets and displayed twice for each block. Forty images were the fillers and displayed once. Eight of the targets were presented with a long distance of 100–109 images between an image and its repeat. Thirty-two of the targets were presented with a short distance of 2–5 images.

## 3. Results

Our test battery includes multiple parameters in each task, and each participant engages in all tasks. This experimental design enables us to evaluate cognitive tasks either within each task in detail or across different diverse tasks. In terms of cognitive test batteries, the evaluation contributes to understanding the effect of parameters on each task, as multiple cognitive abilities can mediate each task. In terms of cognitive mechanism understanding, it contributes to connecting the understanding of each task with other tasks' performances.

This section first describes the basic performance of our cognitive test battery to confirm whether our stimulus manipulation can capture diverse individual differences and how parameter differences affect the performance. Then, as in other cognitive test batteries, we summarize the reliability and validity on the tasks. We evaluate the reliability of our test battery by analyzing the cross-day performance consistency. In Section 3.3, we analyze the latent cognitive factor and evaluate the validity of our tasks to measure human attention and memory. We discuss our latent factors compared with the previous works in cognitive test batteries and cognitive sciences in Section 4 to clarify the position of our test battery.

### 3.1. Basic Performance

**Figure 5:**
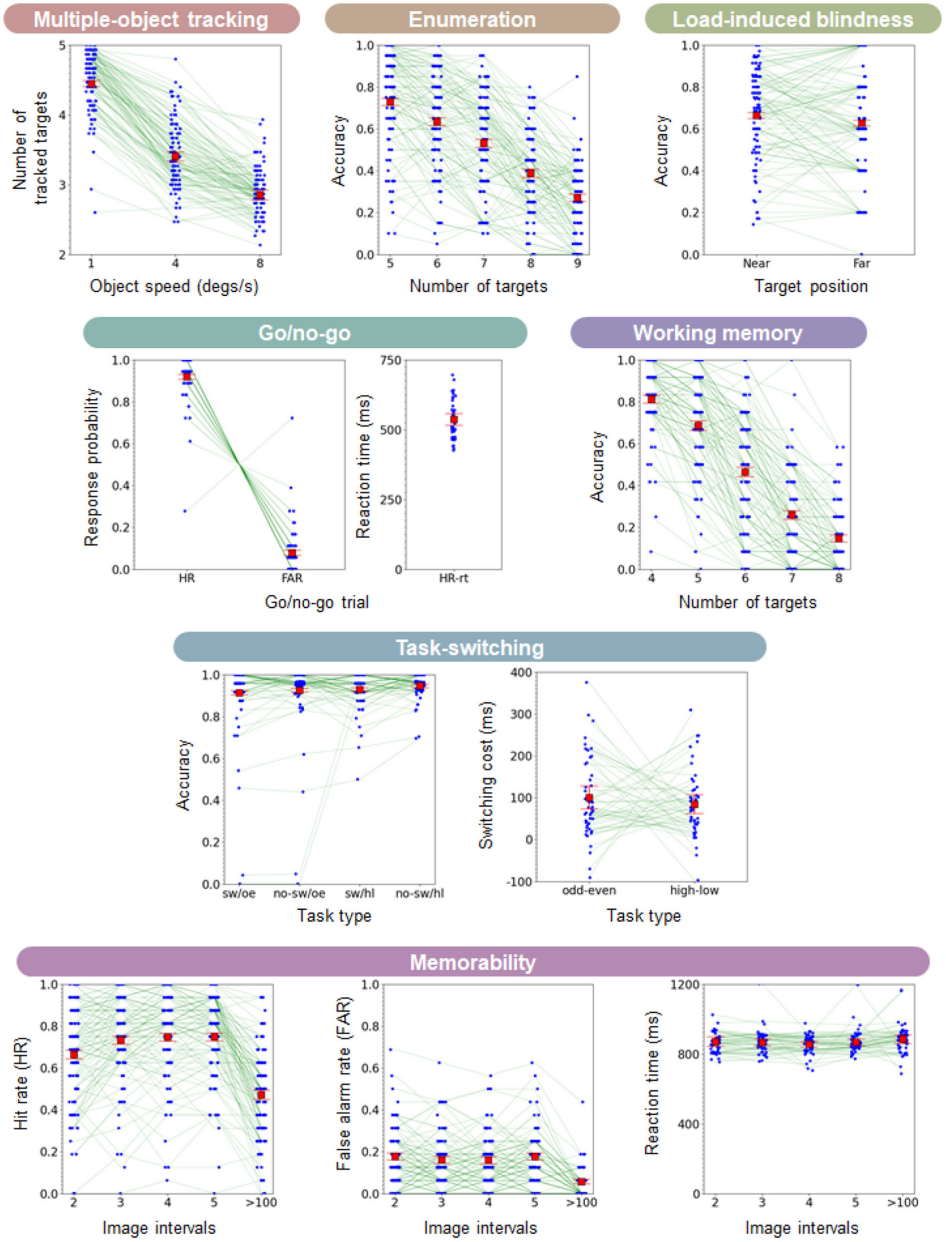
Results of all tasks. The response probability or reaction time is shown for each task. The horizontal axis of each panel indicates the stimulus conditions. The small blue circle depicts the individual performance. The thin green line connects each individual performance across different stimulus parameters. The red square and error bars show the mean probability and 95% credible intervals calculated from Bayesian statistical simulation.

Figure 5 shows the results of basic performances for each task. Different panels show different tasks. Blue circles of each panel indicate the individual performance of the response probability or reaction time for each task condition. The thin green line connects each individual performance across different stimulus parameters. We analyzed the data using Bayesian statistical methods to estimate the mean parameters (accuracy and reaction time) and their 95% credible intervals, shown in the red squares and error bars in Figure 5 (Andrews and Baguley, 2013; Makowski et al., 2019). Our main motivations to use bayesian inference were the access to credible intervals and to the a posteriori distribution (not only to a point estimate) (Kruschke, 2021). We performed the model inference by Hamiltonian Monte Carlo with the NUTS sampler using PyStan. The simulation parameters of the chain and the iteration were 4 and 10,000, respectively. We estimated the accuracy parameter per task and per condition for each participant by using the binomial distribution as the likelihood and with the uniform distribution on the interval [0, 1] as prior for the probability of success per trial. The estimated accuracy was averaged across observers for each sampling and calculated the 2.5 and 97.5% percentiles of the distribution (i.e., 95% credible interval). For the reaction time estimation, we estimated the parameter per task and per condition for each participant by using the normal distribution as the likelihood and the uniform distribution on the interval [0, 1,000] as a prior. As in the accuracy estimation, we estimated the averaged mean reaction times and their 95% credible intervals.

We calculated the correct response probability (accuracy) of each stimulus condition for the enumeration, the load-induced blindness, the multiple-object tracking, and the working memory tasks. The accuracy for the multiple-object tracking corresponds to how many objects participants could track, as for the ordinate of the multiple-object tracking tasks in Figure 5. For the memorability and go/no-go tasks, we defined the hit rate (HR) and the false alarm rate (FAR) according to the previous works. The HR for the memorability indicated the correct response probability for the images presented for the second time. The HR for the go/no-go task indicated the correct response probability for the go trials. The FAR for the memorability and go/no-go tasks meant the wrong response probability for the images presented for the first time and the wrong response probability for the no-go trials, respectively. We also evaluated the reaction time (RT) for the trial on which observers correctly responded. For the task-switching task, we used the switching cost metric in addition to the accuracy of the switch and non-switch trials. The switching cost refers to the reaction time difference between the switch and non-switch trials. The positive switching cost indicates that participants took more cognitive load for the switching condition. We evaluated this either the odd/even or large/small condition.

Since a cognitive test battery aims to measure personalized cognitive state, it needs to cover diverse individual differences. Our results showed the large individual difference in the accuracy on the enumeration, the multiple-object-tracking, the load-induced blindness, the working memory, and the memorability tasks (blue circles in Figure 5). In addition, the relative individual performance was not consistent across different stimulus conditions for some tasks. For instance, the individual trends for the enumeration and working memory tasks, depicted by the green lines in Figure 5, show complex interactions depending on the stimulus parameters.

For the go/no-go and the task-switching tasks, the response probability of HR/FAR and the response accuracy were saturated on most participants, but the reaction time and the switching cost time showed large individual differences, respectively.

Although our results showed large individual differences, the overall performance across participants, shown in the red squares and error bars in Figure 5, changed with the stimulus condition on each task, consistent with previous works. The task performance on the enumeration task decreased as the target number increased (Trick and Pylyshyn, 1993). For the multiple-object-tracking, the task accuracy and the averaged tracking number decreased as the object speed increased (Bettencourt and Somers, 2009). For the load-induced blindness task, most participants missed the target detection regardless of the condition (near or far) (Eayrs and Lavie, 2018). The switching cost was positive for large/small or odd/even task type (Monsell et al., 2003). The working memory task performance was also decreased with the target number participants remembered (Berch et al., 1998). For the memorability task, the HR was decreased when the target interval was long (>100) compared to when the target interval was short (Khosla et al., 2015).

### 3.2. Reliability Across Days

We calculated the reliability across 2 experiment days. The purpose of the analysis is to understand how repeating the set of tasks affects the performance. When the test battery is used as the pre/post assessments of cognitive training, one needs to understand the reference performance of repeating the tasks without training to evaluate how much the training improves cognitive ability.

**Figure 6:**
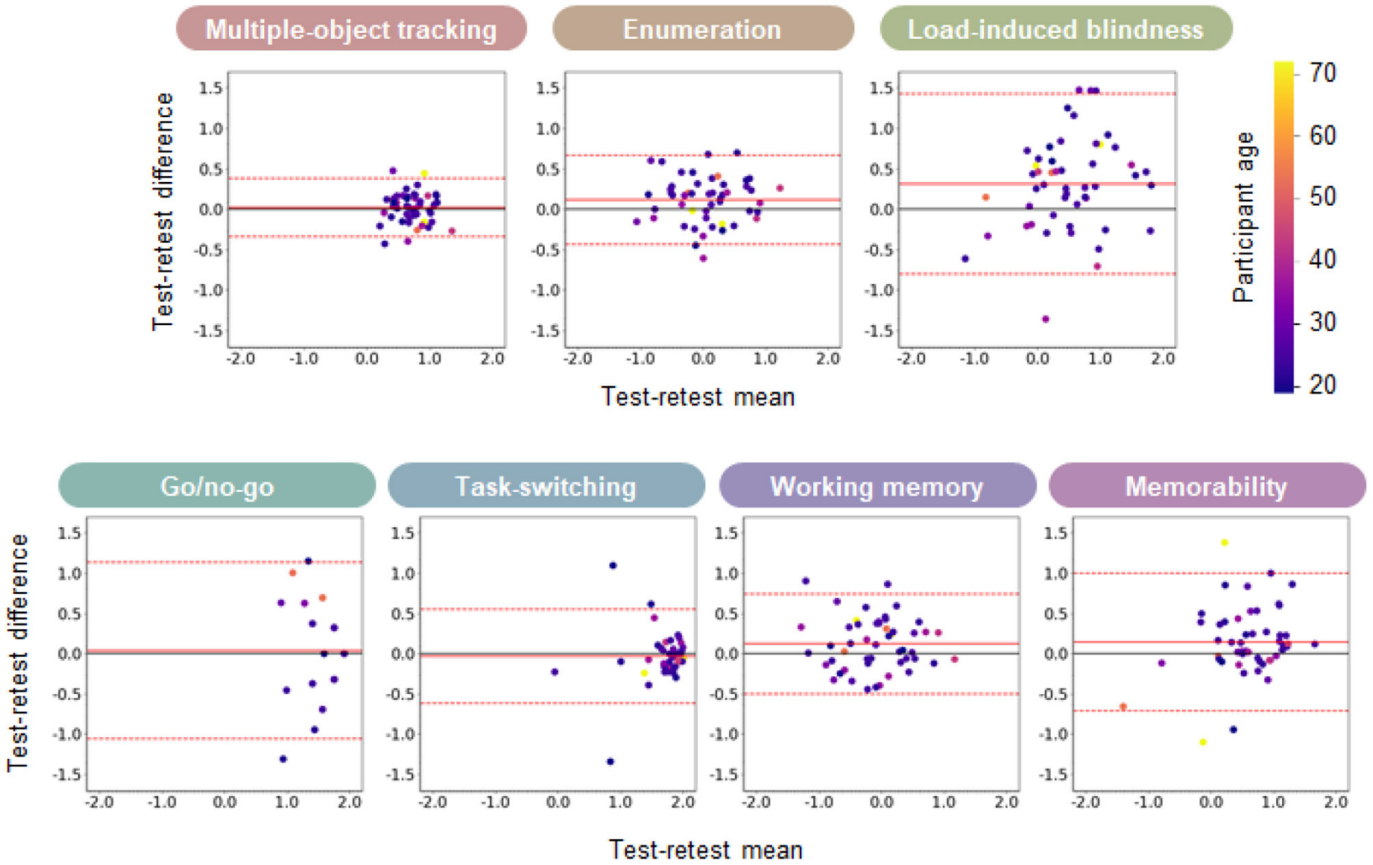
Bland-Altman plot for the accuracy data. Each participant's performance difference was plotted as a function of the mean performance of the 2 days. Different colors indicate different participant ages. The accuracy data is converted to the inverse normal cumulative distribution function, as in the latent factor analysis. The positive difference means that the second day performance is better than the first day. The solid red line indicates the mean difference across participants, while the dashed red line indicates the ±1.96 SD of the differences.

Each participant engaged in the same tasks for 2 days in our experiment. We evaluated the test-retest reliability across the days with two traditional metrics in the cognitive test battery literature and one analysis based on Bayesian statistics. First, we calculated the performance correlation between the days. The Pearson's correlation coefficients (r) of the accuracy for each task were as follows: (1) multiple-object tracking; 0.89, (2) enumeration; 0.81, (3) load-induced blindness; 0.52, (4) go/no-go; 0.95, (5) task-switching; 0.56, (6) working memory; 0.89, and (7) memorability; 0.66. Second, we conducted the Bland-Altman analysis across days (Bland and Altman, 1986; Figure 6). The Bland-Altman plot visualizes the performance differences across the days as a function of the mean performance. Each plot indicates each participant colored with age. We averaged each participant's accuracy across different conditions in each task. Results showed that some participants showed performance improvement (the positive value in the test-retest difference), but we could not observe a clear trend of age on the improvement.

**Figure 7:**
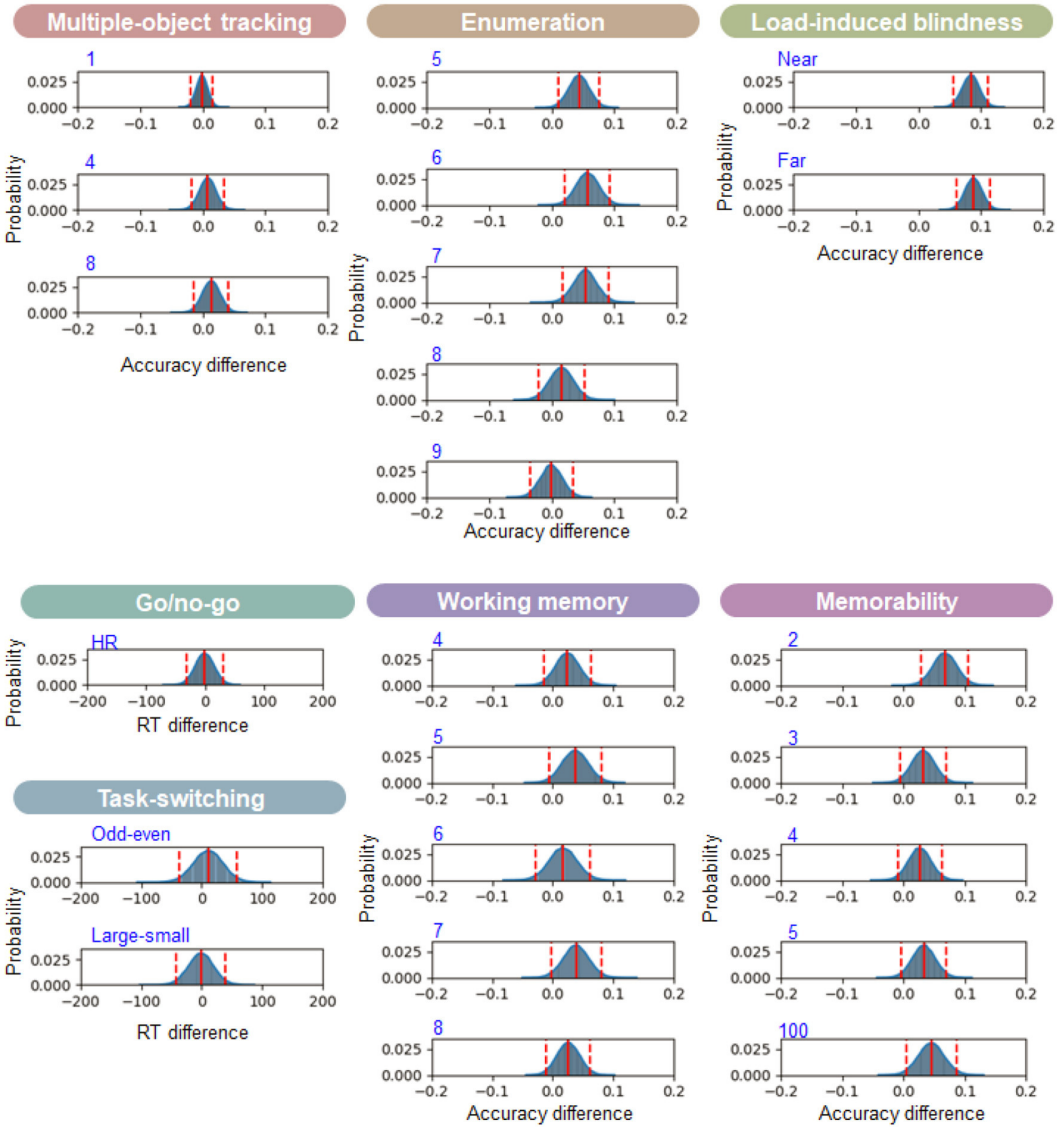
Cross-day performance difference. Each panel shows the probability density of the posterior distribution difference between the first and second-day performance (Day 1–Day 2 for the RT difference and Day 2–Day 1 for the accuracy difference). The positive value means the better performance in Day 2 for either RT or accuracy differences. The vertical red solid and dotted lines indicate the mean and 95% credible intervals, respectively. The condition names put on the left-top for each panel with blue color correspond to the ones shown as the abscissa in Figure 5. We show here cross-day performance for the parameters used in the latent factor analysis.

Next, to evaluate these test-retest effects statistically, we analyzed the posterior distribution differences of test-retest performances using the Bayesian analysis. Figure 7 shows the posterior distribution differences between the first and second-day performance. We first estimated the posterior distributions of 10,000 samples of each day by Bayesian statistical methods described above and took the difference of the 2 days. We subtracted Day 1 from Day 2 for the accuracy distribution and Day 2 from Day 1 for the reaction time distribution to make the training effect positive. We focused on how much the mean difference of each distribution and the 95% credible interval (i.e., highest density interval) deviated from the zero of each difference distribution. The more the distribution deviates to the positive direction, the better the second-day performance is than the first-day one. Results showed that the 95% credible intervals included the no difference point for 32 out of 39 conditions. The credible interval deviated from the point for the long interval and one short-interval condition in the memorability task, the small number conditions in the enumeration task, and the near and far conditions in the load-induced blindness task. Even for these conditions, the mean distribution difference, indicating the effect size of repeating the task, was relatively small (<0.1 probability e.g., <2 trials per session for load-induced and enumeration task). These findings suggest that the task performance does not improve simply by repeating the tasks twice, and therefore, the test battery is appropriate for the pre/post assessment for cognitive training to evaluate how much the training was effective for participants' cognitive ability.

### 3.3. Latent Factor Analysis

Our cognitive tasks captured the large individual difference, but there remains a question about what internal cognitive factors mediate these differences. To explore the factors, we conducted the latent factor analysis, as in cognitive test battery validation (Vermeent et al., 2020). We first transformed the probability data using the inverse normal cumulative distribution function to deal with it for continuous decompositions. We converted the zero and one probability according to the total trial number (i.e., corrected the zero value to 1/2N and the one value to 1-(1/2N), where N is the total trial number) to avoid the infinity value of the transformation (Macmillan and Kaplan, 1985). After data normalization, subtracting variable means from each observation and scaling it using variable standard deviations, we applied principal component analysis (PCA) to the data. We used 23 variables extracted from the task conditions, shown in Figure 7, and 100 participants' data of the first and second days for observations.

**Figure 8:**
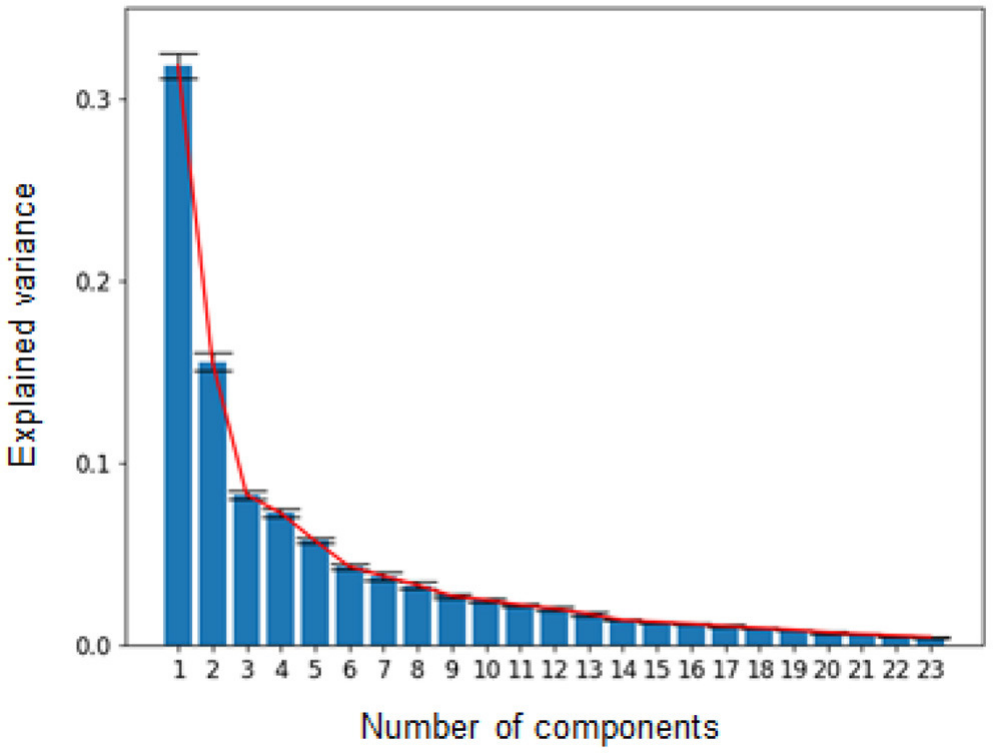
Scree plot of the latent factor analysis.

**Figure 9:**
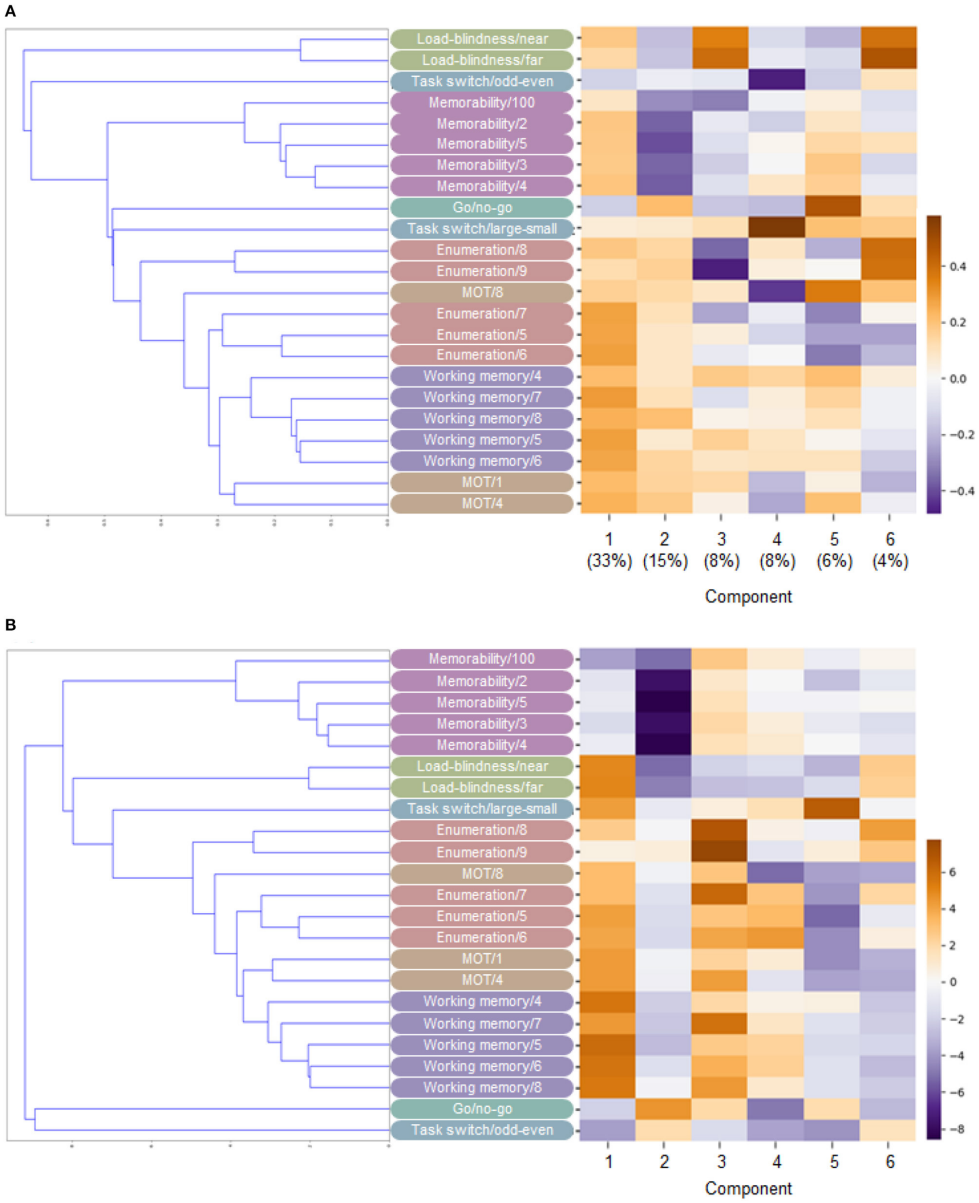
The hierarchical clustering and the loading in **(A)** PCA and **(B)** ICA. The components in PCA are numbered in order of the magnitude of the explained variance, as in the legend. The component order in ICA is arbitrary because the analysis does not have the priority of the order.

Figure 8 shows the explained variances in PCA as a function of the number of components. Based on the plot, we extracted the six components because the cut point shows an “elbow” point (Nguyen and Holmes, 2019). When including the six components, the total explained variance was over 70%, and each point after six only explains the variance of <4%. By using the six factors, Figure 9 visualizes the loading of each component and the hierarchical clustering based on the latent factor similarity between different task conditions. The first component showed negative for reaction data of the go/no-go and task-switching tasks and positive for the accuracy data for the other tasks. While the smaller value in the reaction time means a fast (better) response, the larger value in the accuracy means better performance. Therefore, the first component can be associated with a general ability factor, the shared ability across different cognitive tasks to solve them (Steyvers and Schafer, 2020).

The latent factors after the first one in PCA are constrained by the orthogonality of the input parameters. To interpret the factors conservatively, we also conducted the independent component analysis (ICA), where the latent factor orthogonality is not constrained by the input parameters. We used the FastICA implemented in the python scikit-learn library with the six parameters. The results showed that the hierarchical clusterings of the ICA were similar to PCA (Figure 9). It is noteworthy that the different conditions within the same task were not always clustered in near categories. For instance, these hierarchical clustering analyses showed that the larger target numbers in the enumeration tasks (8 and 9) were separated from the smaller numbers. Similar trends to this separation were observed in the MOT task.

**Figure 10:**
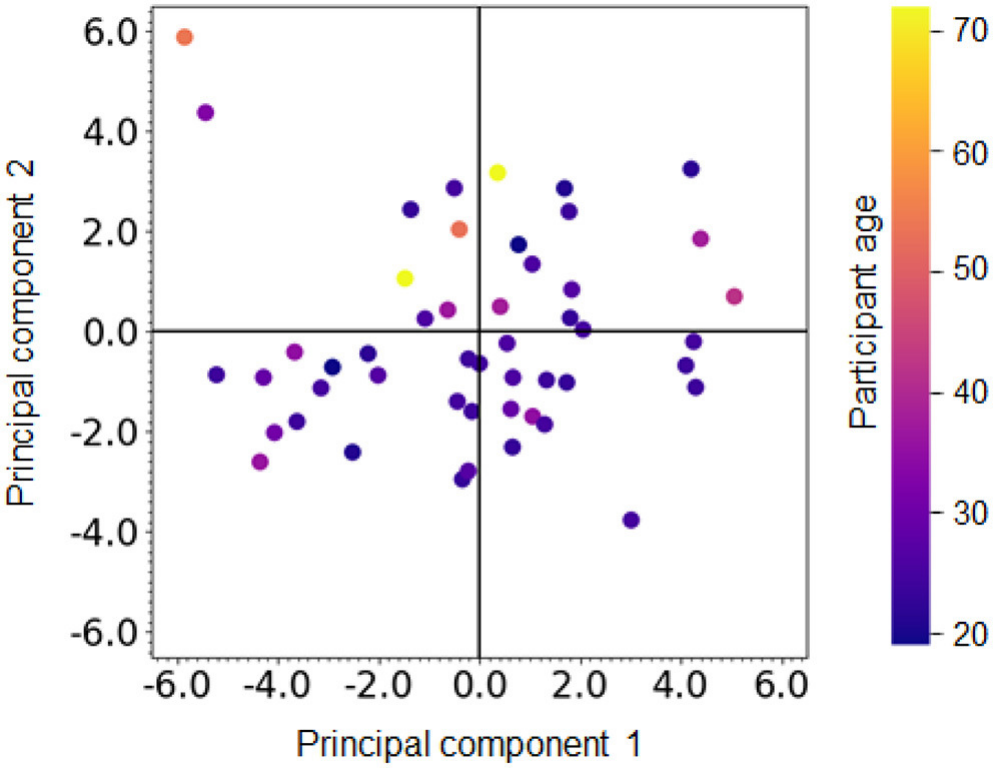
Individual data for PCA components 1 and 2. Different colors indicate different participant ages.

For either PCA or ICA, the loading results showed the factors to which the memorability tasks contributed, correlated with the load-induced blindness tasks and the large numbers of the enumeration task. For instance, components 1 and 2 in PCA have the loading of the same sign from the memorability tasks and the load-induced blindness tasks, and similar trends can be found in component 2 in ICA (Figure 9). Besides, component 3 in PCA and component 3 in ICA shows the correlated loading from the long-interval memorability task and the large numbers of the enumeration task (Figure 9). Besides, the loading results for ICA showed the factors to which the MOT contributed, correlated with the enumeration, the load-induced blindness, and the working memory tasks. For example, the MOT, the enumeration, the load-induced blindness, and the working memory tasks contributed to component 1 (Figure 9B). Also, the MOT, the enumeration, the working memory, the go/no-go, and the memorability tasks contributed to component 3 (Figure 9B). Figure 10 showed the individual participant distribution of the first and second PCA latent components. Each plot is colored according to each participant's age. All participants' data and their basic attributes (i.e., age) are shared in our repository for users to review their future works.

## 4. Discussion

The objective of the study is to create and evaluate the cognitive test battery to measure diverse human attention and memory. The test battery includes seven cognitive tasks: multiple-object tracking, enumeration, load-induced blindness, go/no-go, task-switching, working memory, and memorability. The results of the basic performance show systematic shifts according to the task difficulty and suggest that our test battery covers diverse individual differences. The reliability analysis shows that the task performance across different days is highly similar to each other. Cross-day reliability is essential to use this test battery for cognitive training because learners repeatedly engage in the cognitive assessment before/after their training. The reliability analysis shows the reference performance when the tasks are repeated twice without training. In addition, the latent factor analysis showed what internal cognitive factors mediate the individual differences. Specifically, the results suggest that a general ability across all tasks and some task-specific ability underlie the cognitive test battery performance.

These latent factors are consistent with the previous behavioral and neurological findings in cognitive science literature. Many works with a large-scale cognitive task have also reported the shared ability across the visual attention and memory tasks (Steyvers and Schafer, 2020; Panichello and Buschman, 2021). For instance, Steyvers and Schafer (2020) investigated the behavioral performance with a large-scale cognitive test and analyzed the latent factors using probabilistic PCA. They found that a general ability factor mediates across all tasks, including visual tasks like ours. Panichello and Buschman (2021) recently suggest from their neurological investigation that the prefrontal cortex works as a domain-general controller for attention and memory tasks. In addition to the general ability, the domain-specific components are also discussed in previous findings (Miyake et al., 2000; Friedman et al., 2008; Larrabee, 2015; Nakai and Nishimoto, 2020; Vermeent et al., 2020; Panichello and Buschman, 2021). For instance, Friedman et al. (2008) investigated latent factor analysis of executive function and suggested that updating and shifting function mediates the task performance in addition to a common cognitive factor. The working memory and the visual-spatial processing are separate but related factors (Larrabee, 2015), and the tasks related to these factors could be separately represented in the brain, in addition to overlapped common representation (LaBar et al., 1999).

In the cognitive test battery context, the domain-specific latent components are tightly connected to specific tasks. For instance, the working memory factor loads to only span tasks in the validation study of a computerized cognitive test battery (Vermeent et al., 2020). In contrast, most of our latent factors were not task-specific, e.g., we did not see the factor only affecting our spatial span task and we found the multiple factors affecting the same task. In addition, it is noteworthy that our results are consistent with previous cognitive science works. For instance, some studies have shown that the working memory performance is related to the MOT (Allen et al., 2006; Lapierre et al., 2017), consistent with our results about the components 1 and 3 of the ICA analysis (Figure 9B). These findings suggest that our latent factors capture more focused cognitive abilities than general cognitive test batteries, overlapped across multiple visual tasks.

MOT tasks are common in the cognitive training literature (Legault and Faubert, 2012; Harris et al., 2020; Vater et al., 2021), as we are also conducting such a training project, and it is important to understand to what extent MOT training effects propagate to various cognitive abilities. Evaluating how various task performance is related to MOT abilities in our cognitive test battery contributes to understanding such training transfer in cognitive training works. Our latent factor analysis showed that the general cognitive ability meditates the MOT performance, including other tasks. Furthermore, consistent with previous works, we found the latent factors contributing to the MOT and the enumeration (Figure 9B, components 1 and 3, Green and Bavelier, 2006b), the MOT and the load-induced blindness (Figure 9B, component 1, Eayrs and Lavie, 2018), and the MOT and the working memory (Figure 9B, components 1 and 3, Allen et al., 2006; Lapierre et al., 2017).

The memorability task has been originally suggested in the computer vision literature, and it is not clear about the relationship with classic cognitive tasks. Previous studies have mainly investigated the task in terms of intrinsic image factors driving humans' image memorizing. However, it has also been shown that cognitive factors mediate the task, especially for the intermediate difficulty we used in our memorability test. Some brain imaging and neurophysiological studies also suggested the neural basis of cognitive contributions (Bainbridge and Rissman, 2018; Jaegle et al., 2019; Mohsenzadeh et al., 2019). Specifically, Mohsenzadeh et al. (2019) used a high-resolution-spatiotemporal brain imaging technique with combining fMRI and MEG measurements and recorded the brain activity during the memorability task. They compared brain responses between high and low memorability images and showed that both early visual processing and later cognitive processing mediates the difference between high and low memorability. The present finding can contribute to understanding these processing. Our latent factor analysis showed that the factors including the memorability task are mainly related to the accuracy of the enumeration task with higher difficulty and load-induced blindness task. It has been suggested that missing the target in the load-induced blindness and enumeration is due to inattentional blindness over perceptual capacity and can be a different process from the working memory ability (Bredemeier and Simons, 2012; Eayrs and Lavie, 2018). Our finding suggests that the cognitive processing mediating the memorability task is related to missing the intrinsic image factors in an image due to the inattentional blindness, rather than failure of keeping image contents using working memory ability. This finding is also consistent with the previous result that the memorability performance depends on the eye-gaze position.

Unlike conventional cognitive test batteries, we did not extract a single threshold or slope of a psychometric function for each task but used multiple stimulus parameters' performance for the latent factor analysis. When researchers measure a single threshold for a specific stimulus direction, they implicitly assume that a single cognitive mechanism mediates the task along with the stimulus parameters they controlled. In other words, they assume that participants with the threshold of better performance are superior in a specific cognitive ability. However, this is not always the case if multiple visual mechanisms mediate the task dimension. Consistent with the notion, our latent factor analysis showed that the identical stimulus parameter is not always categorized in the same cluster (Figure 9). Also, the basic performance results showed that the individual trend is highly complex on each task (green lines in Figure 5). The finding suggests that complex interaction lies on the cognitive mechanisms depending on stimulus parameters even in the same task.

We determined the stimuli and procedure of our cognitive tasks by following previous works about cognitive mechanisms of attention and memory. These works tend to overlook individual differences presumably due to small numbers of participant sampling, but our study showed diverse performance differences across individuals for all tasks. For instance, in the task-switching task, the switching cost largely depends on individuals. For some participants, the difference between switching and non-switching trials is more than 200 ms on average, but there are few differences for other participants. When researchers investigate cognitive ability using one specific task, large individual differences are unknown factors making interpretation difficult. However, as in our latent factor analysis, when the same participants engage in multiple tasks, the large individual difference in one task can be a clue for understanding cognitive mechanisms for another task. This notion suggests that our test battery can also be used for the investigation of cognitive mechanisms as a benchmark evaluation of each participant. Some experiments for cognitive mechanism understanding are hard to collect many participants, e.g., brain imaging experiments. If researchers conduct a new investigation with our test battery, the individual differences in the new experiment can be more understandable.

One limitation of our investigation is that we do not strictly control the observer attributes when recruiting participants. One typical attribute affecting cognitive performance is the age of participants. Aging affects various aspects of cognitive abilities. For instance, it has been shown that the capacity of tracking objects in MOT tasks decreases for older participants (Trick and Pylyshyn, 1993; Sekuler et al., 2008; Legault et al., 2013). Legault et al. (2013) used a 3D MOT task, called the Cave Automatic Virtual Environment (CAVE), and showed that healthy older adults have lower tracking ability than younger adults, but that training with a 3D MOT task improves the tracking ability of healthy older adults in a similar learning function to younger adults. In addition to MOT, other cognitive tasks such as visuospatial attention (Greenwood et al., 1993; Curran et al., 2001) or working memory (Salthouse, 1994) depend on the age of participants. Furthermore, it has been known that other observer attributes such as the level of expertise in sports (Faubert, 2013) or gaming (Green and Bavelier, 2006a; Benoit et al., 2020) affect cognitive abilities. One needs to separate the participant group according to the targeting attribute to investigate the effect of each attribute on cognitive performance. Although our investigation does not control the population, we analyzed how the performance of different age participants is distributed in our cognitive tasks (Figures 6, 10). Further investigation is needed to elucidate the effect of observer attributes.

Another limitation is that our online experiment is not strictly controlled in stimulus presentation and response collection compared to laboratory experiments. For instance, the reaction time can be potentially inaccurate due to participants' environment setting because the accuracy depends on the response input device. However, recent studies have suggested that the reaction time measured in web experiments can be comparable with lab experiments (de Leeuw and Motz, 2016; Hilbig, 2016; Armitage and Eerola, 2020). In our experiment, we only measured the reaction time by the keyboard input device, not by the mouse clicking (or touch clicking), and restricting the device contributes to decreasing the measurement distortion (Armitage and Eerola, 2020). Besides, our reaction time data was comparable with previous findings in lab environments. For instance, the reaction time of the memorability task with intermediate memorability scores in a lab experiment is around 900 ms, which is consistent with our current results (Võ et al., 2017). Based on these findings, we believe that using reaction time as a metric for our test battery is acceptable.

In addition, we did not apply the gamma correction according to each monitor's characteristics during our online experiment. One needs a photometer to conduct the gamma correction strictly for each monitor, which cannot be available in online experiments. A way for online experiments is to correct the nonlinearity based on participants' responses using a grating chart, but it could be affected by the quality of participants' responses. We did not apply such user-based correction and presented stimuli without the gamma correction. Previous studies in visual perception and cognition literature have shown that the performance in online experiments can be comparable to that in strictly controlled laboratory experiments for some visual tasks (Bylinskii et al., 2015; Sasaki and Yamada, 2019; Sawayama et al., 2022). For example, the memorability task is conducted both in online and laboratory experiments (Bylinskii et al., 2015). Some studies have suggested that the contrast sensitivity performance could be comparable under sufficient repetition for each condition between online and laboratory experiments (Sasaki and Yamada, 2019) and that suprathreshold contrast discrimination with large contrast differences could be stable across online and laboratory experiments compared to blur discrimination tasks for natural object stimuli (Sawayama et al., 2022). However, the optimal presentation, especially for the Gabor stimuli in the load-induced blindness task, is to use a linearly corrected monitor. The way of presentation can be critical when users conduct our test battery for some populations that have reduced contrast abilities, e.g., older adults. It has been known that contrast sensitivity is worse for older adults than younger adults because aging changes the optical properties of the eyes (Owsley, 2016). When one does not strictly control the stimulus presentation, the effect of such front-end properties can not be evaluated appropriately. Therefore, if users conduct our test battery for such populations in a non-controlled online experiment, they should be extra careful when interpreting the results of the load-induced blindness task to understand whether the obtained performance is due to cognitive abilities or the front-end properties. One additional control for the load-induced blindness in an online experiment might be to conduct a contrast discrimination task without the attention load of the foveal length judgment to confirm whether participants could discriminate the contrast differences without divided attention.

It is noteworthy that we share all source codes and data to conduct the cognitive assessment experiment from our repository (https://github.com/flowersteam/cognitive-testbattery). Not only can users conduct our experiment as we did on their own server, but also they can do it more flexibly. One use-case is to conduct our test battery on a shared server. Another case is to conduct it in the laboratory environment. In this case, users can strictly control the monitor size and viewing distance and run the experiment using a web browser.

## 5. Conclusions

In summary, we suggest an online open-source cognitive test battery including the seven cognitive tasks: multiple-object tracking, enumeration, load-induced blindness, go/no-go, task-switching, working memory, and memorability. Our test battery can flexibly be used either online or in laboratory experiments with a web browser. Our benchmark test shows that it captures diverse individual differences and can evaluate them based on latent cognitive factors. Besides, our results suggest a novel finding that the cognitive factor mediating the memorability task is the ability related to inattentional blindness rather than working memory.

## Data Availability Statement

The datasets presented in this study can be found in online repositories. The names of the repository/repositories and accession number(s) can be found at: https://github.com/flowersteam/cognitive-testbattery.

## Ethics Statement

The studies involving human participants were reviewed and approved by the Operational Committee for the Evaluation of Legal and Ethical Risks (OCELER). The patients/participants provided their written informed consent to participate in this study.

## Author Contributions

MA, MS, P-YO, and HS: conceptualization, methodology, and investigation. MA and MS: software, validation, formal analysis, resources, data curation, and visualization. MA, MS, DM, AD, P-YO, and HS: writing. DM, AD, P-YO, and HS: supervision, project administration, and funding acquisition. All authors have read and agreed to the published version of the manuscript.

## Funding

This work forms part of a thesis project which was sponsored by Onepoint company, Inria Research Institute and French Ministry of Higher Education and Research (CIFRE thesis). The funder was not involved in the study design, collection, analysis, interpretation of data, the writing of this article or the decision to submit it for publication. The only contribution of the funder is to pay the preliminary publishing fees.

## Conflict of Interest

MA, DM, and AD were employed by the company Onepoint when the research was conducted. P-YO is a permanent research director at Inria of the University of Bordeaux (French Public Institute on digital science) and he was employed by MICROSOFT Laboratory at Montreal (Canada) during the academic year 2021–22. The remaining authors declare that the research was conducted in the absence of any commercial or financial relationships that could be construed as a potential conflict of interest.

## Publisher's Note

All claims expressed in this article are solely those of the authors and do not necessarily represent those of their affiliated organizations, or those of the publisher, the editors and the reviewers. Any product that may be evaluated in this article, or claim that may be made by its manufacturer, is not guaranteed or endorsed by the publisher.
